# The Minimally Invasive Star-Shaped Incision Technique and the Linear Incision Technique With Tissue Preservation for Percutaneous Bone Conduction Devices: A Retrospective Cohort Study

**DOI:** 10.3389/fsurg.2022.863997

**Published:** 2022-03-21

**Authors:** Ruben M. Strijbos, Samer Salameh, Aren Bezdjian, Sam J. Daniel, Hans GXM. Thomeer

**Affiliations:** ^1^Department of Otorhinolaryngology and Head and Neck Surgery, University Medical Center Utrecht, Utrecht, Netherlands; ^2^Brain Center UMC Utrecht, Utrecht, Netherlands; ^3^Faculty of Medicine and Health Sciences, McGill University, Montreal, QC, Canada; ^4^Departments of Experimental Surgery and Pediatric Surgery, Faculty of Medicine, McGill University, Montreal, QC, Canada; ^5^Department of Otolaryngology–Head, Neck Surgery, and Pediatric Surgery, Faculty of Medicine, McGill University, The Montreal Children's Hospital, Montreal, QC, Canada

**Keywords:** bone anchored hearing implant, BAHA, bone conduction, star-shaped incision, linear incision

## Abstract

**Purpose:**

To compare intra- and postoperative outcomes between the standard linear incision technique with tissue preservation (LITT-P) and the minimally invasive star-shaped incision (SSI).

**Study Design:**

A retrospective cohort study.

**Methods:**

Primary outcomes evaluated operative time, implant survival, and intra-operative complications. A secondary outcome evaluated soft tissue tolerability assessed by the Holger's classification.

**Results:**

A total of 38 implants were placed (19 LITT-P; 19 SSI). The median and mean surgical duration for the LITT-P group was statistically shorter than the SSI group (*p* = 0.0001). No intra-operative complications were reported for both surgical approaches. Five implants were lost during postoperative follow-up: one in the LITT-P and four in the SSI cohort. Both cohorts showed favorable soft tissue tolerability. Less Holgers 1 and 2 and more Holgers 3 soft tissue reactions were observed after the LITT-P compared to the SSI.

**Conclusion:**

The novel SSI approach could be an alternative option based on the theoretical benefits and found favorable (and similar) soft tissue outcomes. Implant loss and surgical time are aspects to investigate regarding long-term durability and warrant further research.

## Background

Osseointegrated bone conduction devices (BCDs) were first described in the 1970s. Since then, these implantable systems have rehabilitated adults and children with hearing loss with success rates of 90% or higher (1–4). BCDs rely on the transmission of sound *via* bone conduction sensed by the inner ear. These devices are comprised of an external sound processor, which is coupled to a titanium fixture implanted into the skull behind the ear.

The percutaneous BCD has seen many enhancements since the first reported case (1). In the past, skin thinning techniques were advocated to minimize postoperative skin reactions and possibly decrease extrusion rates (2–5). In the last decades, new insights have supported the idea that skin preservation outperforms these older techniques with better results in skin reactions, postoperative pain, operative time, and scarring (6). Since 2010, several groups have reported improved tolerance to percutaneous devices implanted without reduction of the soft tissues surrounding the percutaneous abutment (6). Consequently, nowadays, the most performed surgical approach is the non-skin thinning linear incision technique with soft tissue preservation (LITT-P). In combination with innovations in implant and abutment design, this resulted in low incidences of implant failures, increased overall satisfactory rates, and led to early sound processor coupling protocols to shorten the period of auditory deprivation (5–8).

Recently, so-called “punch only techniques” have been introduced to develop an even more minimally invasive surgical approach (5, 9–11). The minimally invasive ponto surgery (MIPS) technique is such an innovation, developed by Oticon Medical AB (Askim, Sweden), which also promotes BCD placement under local anesthesia (12). This “punch only” approach is conducted in a single-stage procedure that aims to reduce surgical time and variability, reduce the incision scar, and minimize trauma to the bone and soft tissue (13, 14). Nonetheless, some studies reported a higher rate of implant loss after the MIPS (15, 16). It is hypothesized that possible explanations may be angulated insertion or interposing soft tissue as a result of the reduced visibility. Also, the small incision may lead to too much heat generation ergo negative effects on the bone at the implantation site (17). Moreover, the MIPS is reserved for the Oticon Ponto® BCD (16, 18). The aforementioned challenges did lead to the innovation and introduction of the star-shaped incision approach (SSI) in the University Medical Center Utrecht. The SSI approach consists of a single-punch incision, enlarged by three incisions perpendicular to the punch, giving it its “star shape.”

The objective of the current study was to present the novel SSI approach and compare its peri-operative outcomes with the LITT-P approach in a retrospective cohort series.

## Materials and Methods

### Study Design, Population, and Outcome Measures

This was a retrospective cohort study. Data were extracted from a retrospectively collected database of BCD recipients operated between March 2015 and August 2019 with the star-shaped incision (SSI) and the linear incision with soft tissue preservation technique (LITT-P). Only adult patients (18 and above) were included. Extracted data included patient demographics (age at intervention, gender, laterality of implant), comorbidities, operative information (use of anesthesia, surgical time, screw/abutment characteristics, and manufacturer), and postoperative outcomes (soft tissue integrity, complications, and implant survival). The surgical time was defined as the time between the start incision and the end of the surgical procedure. Tolerability of the soft tissue surrounding the implant site was monitored and classified according to the Holger's classification (19). The classification system grades soft tissue reactions at the implant site regarding redness, swelling, moistness, and granulation tissue. The follow-up of the patients was until March 21, 2021.

### Description of Procedures

Two different surgical approaches were used for implantation: linear incision technique with soft tissue preservation (LITT-P) and star-shaped incision technique (SSI). For both surgical approaches, adequate positioning of the implant is according to the international standard (5–6 cm behind the ear canal and around 45–60° in relation to the Frankfurter line). Prior to surgery, the selection of a BCD manufacturer (Baha Cochlear® or Ponto Oticon®) was based on an audiological assessment at an outpatient clinic. Also, the type of anesthesia (local vs. general) was depending on the patient's preference. Skin and soft tissue tolerability was evaluated at every follow-up visit and was categorized using the Holger's classification.

#### Linear Incision Technique With Soft Tissue Preservation (LITT-P)

Implantation through a linear incision without soft tissue reduction as described by Hultcrantz was performed (20). A retroauricular linear incision was made down to the periosteum. Subcutaneous tissue was dissected for exposure and mobilization of the periosteum at the intended implant site. This was followed by the drilling procedure with saline irrigation for cooling, with subsequent widening using a countersink drill as described by Tjellstrom and Granstrom (21). The implant with a mounted abutment was installed. The skin posterior from the incision was mobilized and repositioned over the abutment. Finally, a hole was punched in the skin overlying the abutment. The incision was closed with interrupted sutures. Non-adherent dressing soaked with antibiotic ointment was wrapped around the abutment, and a healing cap was attached.

#### Star-Shaped Incision Approach (SSI)

**Figure 1:**
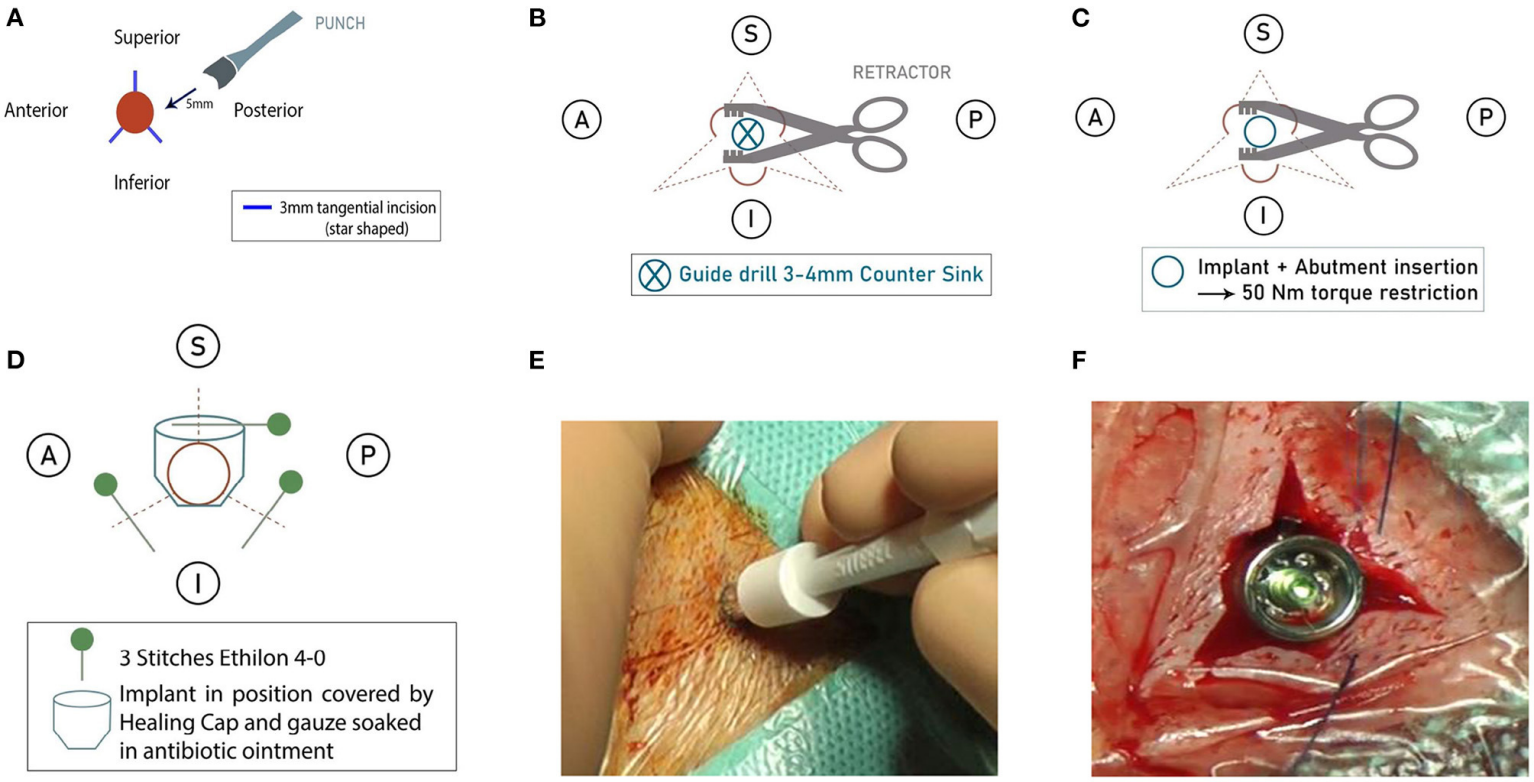
**(A)** Step 1: A punch followed by three perpendicular incisions. **(B)** Step 2: Use of retractors to expose the bone (an implant site). **(C)** Step 3: Drilling and placement of implant and attached abutment. **(D)** Step 4: Closing of incisions, antibiotic wound dressing with a healing cap. **(E)** Image: Punch incision on the preoperative drawn position. **(F)** Image: Perpendicular incisions closed with three cutaneous stitches (Ethilon 4-0).

The SSI approach was performed, under either local or general anesthesia, as follows: Following determination of the adequate implant position, the sterile field was acquired with regular draping. A 5-mm punch incision was performed on the preoperative-drawn position as a through-and-through incision onto the cortical bone. This punch incision was enlarged by three incisions perpendicular to the original punch: around 3-mm length in three directions with equal distance to each other, creating a star shape. Soft tissue with periosteum was deviated, and one retractor was placed exposing the bone adequately. Subsequently, regular drilling was performed (guide drill, 3–4 mm, countersink), and the implant was positioned with torque restriction, 50 Nm. The perpendicular incisions were closed with three cutaneous stitches (Ethilon 4-0), and antibiotic wound dressing (gauze with tetracycline ointment) with a healing cap was applied. This four-step novel approach developed by the University Medical Center Utrecht is summarized in Figure 1.

### Statistical Analysis

The distribution of continuous data was presented using graphs with error bars that allowed for a comparison of the differences between the means within the groups. When the data were not distributed normally, medians and ranges were added to present the continuous data adequately (age). Categorical data were summarized using percentages (gender, indication for surgery, skin tolerability). Differences in baseline characteristics between the cohorts and peri-operative outcomes were tested using the non-parametric, independent-sample Mann–Whitney U tests for continuous variables and the Fisher's exact tests for categorical variables. *P*-values below 0.05 were considered statistically significant.

### Ethics

According to the Dutch Central Committee on Research Involving Human Subjects (CCMO), this study did not require formal approval of the Netherlands Medical Research Involving Human Subjects Act.

## Results

### Patient Characteristics

A total of 38 BCDs were implanted in 37 patients (one patient had an implantation bilaterally with the SSI approach). The implantation was performed with SSI in 19 BCDs (17 Oticon and 2 Cochlear) and LITT-P in 19 BCDs (15 Oticon and 4 Cochlear). The mean age was 57.28 and 60.5 years in the SSI and LITT-P group, respectively, and most patients presented with conductive hearing loss (11 in SSI and 9 in LITT-P). In the SSI cohort, 8 patients (44.44 %) opted for local anesthesia in comparison with 12 patients (63.16 %) in the LITT-P cohort. An overview of the patient characteristics is shown in Table 1. There were (except for choice of anesthesia) no notable differences between the groups. A detailed summary of patient comorbidities is provided in Table 2.

**Table 1:** Patient characteristics.

**Patient characteristics**	**SSI**	**LITT-P**
**N, implants**	19	19
**N, patients**	18	19
**Gender** (# patients)		
Male	9; 10 implants	10
Female	9	9
Not Specified	0	0
**Mean age at surgery [Range] (years)**	57.28 [29–80]	56.84 [21–95]
**Median age at surgery (years)**	60.5	59
**Type of hearing loss**(# patients)		
CHL	11	9
SNHL	0	0
Mixed HL	3	6
Other	1	1
Not Specified	3	3
**Implant laterality**		
Left	10	8
Right	7	11
Bilateral	1	0
**Choice of anesthesia**		
General	10	7
Local	8	12

*LITT-P, Linear Incision Technique with Soft Tissue Preservation; MIPS, Minimally Invasive Ponto Surgery; SSI, Star-Shaped Incision; HL, Hearing Loss; CHL, Conductive Hearing Loss; SNHL, Sensorineural Hearing Loss*.

**Table 2:** Comorbidities.

**Comorbidities**	**SSI(# patients)**	**LITT-P(# patients)**
**Cardiovascular disease**	5	6
HTN	2	4
PAOD	1	1
MI	1	1
Arrythmia	2	0
**Respiratory disease**	4	6
COPD	2	3
OSA	0	2
**Diabetes**	1	2
**Chronic Rhinosinusitis**	0	3
**Down Syndrome**	1	1
**Rheumatoid Arthritis**	1	1
**Ulcerative Colitis**	1	1
**Psoriasis**	2	0
**Malignancy**	2	0

*HTN, Hypertension; PAOD, Peripheral Arterial Occlusive Disease; MI, Myocardial Infarction; COPD, Chronic Obstructive Pulmonary Disease; OSA, Obstructive Sleep Apnea*.

### Primary Outcomes: Surgical Time, Intra-Operative Complications, and Implant Loss

The mean surgical time in the LITT-P cohort was 18.25 min (median, 18 min; range, 5–41 min) vs. 23.64 min for the SSI cohort (median, 22 min; range, 9–46 min). The shortest surgery was performed in 5 min in a patient undergoing the LITT-P technique with local anesthesia and sedation.

No intra-operative complications were reported for both surgical approaches. In the post-operative follow-up, five implants were lost to extrusion with a distribution of one in the LITT-P and four in the SSI group. The single case of extrusion in the LITT-P cohort was not preceded by any signs of pain, inflammation, or trauma and occurred 2 days after the surgical procedure. Multiple comorbidities were noted in this patient, including heart failure, myocardial infarction, COPD, recurrent pneumonia, and multiple previous tympanoplasty surgeries. Regarding the four cases of extrusion in the SSI cohort, there was one case caused by trauma, one preceded by signs of infection in combination with psoriasis and one followed by pain around the implant. The final case was not preceded by any signs of pain, inflammation, or trauma.

### Secondary Outcome: Skin Tolerability

There were a total 71 observations on soft tissue tolerability. The mean follow-up period was 88.42 weeks (range, 2–280 weeks) and 86.47 weeks (range: 1–205 weeks) for the SSI and LITT-P, respectively. Holgers Grade 2 was observed to a lesser extent than Holgers Grade 1 in both cohorts, with 16 reports in SSI-implanted patients and 10 reports in LITT-P-implanted patients. After long-term follow-up, there were seven observations of a soft tissue reaction Holgers Grade 3, with 3 registrations in SSI-implanted patients and 4 registrations in LITT-P-implanted patients. No Holgers Grade 4 reaction was reported in either cohort (Table 3).

**Table 3:** Skin reactions and postoperative follow-up.

**Holger's classification**	**SSI**	**LITT-P**
Grade 1: light redness and slight swelling	21	17
Grade 2: redness and swelling	16	10
Grade 3: redness, swelling, moistness, and slight granulation tissue	3	4
Grade 4: redness, swelling, moistness, granulation tissue, and infection	0	0
**Postoperative follow-up**		
Mean postoperative follow-up period (weeks)	88.42	86.47
Median [Range] postoperative follow-up period (weeks)	42[2-280]	85[1-250]

## Discussion

Two surgical approaches, the star-shaped incision technique (SSI) and the linear incision technique with tissue preservation (LITT-P) were compared in this retrospective cohort study. There were no intra-operative complications, and the surgical time of the LITT-P was significantly shorter. Both techniques showed beneficial soft tissue outcomes with minimal Holgers Grade 3 observations and no Holgers Grade 4 registration. A total of five implants were lost with one implant in the LITT-P and four implants in the SSI cohort during total follow-up.

The percutaneous bone conduction devices (BCD) are a successful option in the hearing rehabilitation of individuals who meet the eligibility criteria. As stated earlier, the BCD has seen many improvements, mostly involving the design of the implants and the surgical approaches to implantation. These innovations aimed to improve implant stability, with sufficient surgical (albeit minimal) exposure and overall satisfaction while reducing surgical variability, post-operative complication, and operative time. Nowadays, the LITT-P technique is the most used surgical approach, and current research focuses on refinement of minimally invasive techniques like the MIPS and the SSI developed in our center.

The development of the SSI was inspired by the challenge of a standardized and more minimally invasive procedure than the LITT-P, but with more visual exposure of the bone than other punch-only techniques. The idea is that a lack of visibility leads to inserted angulation, interfering soft tissue, and excessive heat generation, which potentially results in less osseointegration/implant stability. More exposure of the bone may overcome these concerns because of the direct/perfect visualization and exposition for the surgeon during implant placement. Moreover, another advantage of the SSI compared to the LITT-P might be the lack of traction on the skin, as the skin is closed without any tension (the implant should reside exactly in the middle of the SSI). Our experience shows that the SSI technique can be performed under local or general anesthesia, and it can be used for both manufacturers (Oticon and Cochlear). Nevertheless, disadvantages of the SSI may include wound healing problems after the procedure, especially after release of stitches where the skin around the implant might become dehiscent as more space has been created around the implant during the procedure compared to, for example, the LITT-P. Consequently, the skin might not be completely healed after 6–8 days post-surgery. In the context of evidence-based surgery, the current retrospective study was initiated to assess the SSI in clinical practice.

In our study, there was a significant difference in surgical duration. The SSI had a longer surgical time than the LITT-P. It is important to interpret this outcome in the context of the learning curve for the surgeon for the SSI. In addition, there is time required to get acquainted with the operating room and surgical team performing this new technique. Moreover, we must emphasize the role of the learning curve of the surgeon in contributing to the variability in operative duration in both cohorts, firstly by gradually improving the novel SSI approach, as well as switching and adapting to the LITT-P approach later in the study. Furthermore, although all procedures are performed by the same surgeon, there are case-specific characteristics, including patient age, comorbidities, cranial anatomy, type of implant, and type of intraoperative anesthesia, that provide numerous causes for case-by-case variability in duration of surgery.

Another important finding is the relatively high rate of implant loss in our study population: one in the LITT-P but four in the SSI cohort. A possible explanation could be that the SSI technique is new and needs adaptations and perfections in surgical handling, as in all new implemented techniques. As described in the outline of the surgical SSI procedure, the visibility is better compared to punch-only techniques like the MIPS, providing a nice overview of the bone to be drilled. Also, there is no need for possible excessive heat generation, with its adverse events, such as osteocytic degeneration, fibrosis, increased osteoclastic activity, and necrosis, as similar exposition of the bone is provided (compared to the LITT-P). The philosophy of a star shape instead of a linear incision is the more equal and 360° distribution of skin traction during postoperative healing. Moreover, no skin mobilization is necessary as is sometimes the case during the LITT-P technique where skin at the posterior side of the incision is pulled over the BCD in place. Finally, it is worth mentioning that one patient in the SSI cohort lost his implant due to trauma and two patients had risk factors in implant-related problems: one patient in the SSI had dermatological disease psoriasis, and one patient in the LITT-P had extensive cardiovascular comorbidity. Hygienic and lifestyle differences could also be attributed to changes in implant stability, but this was not monitored.

Overall, there were favorable soft tissue outcomes. The LITT-P cohort had lower soft tissue complications as assessed by the Holgers classification but more Holgers Grade 3 reactions than SSI. Therefore, neither of the investigated techniques outperformed each other in terms of skin complications. Soft tissue reactions are commonly associated with, for example, a delay in processor loading time (22, 23) and recurrent outpatient visits, which may affect patients' quality of life negatively.

This study has some limitation. The small sample sizes of the SSI and LITT-P cohorts must be highlighted. Also, the retrospective nature and the lack of randomization, for example, to the type of technique applied (SSI vs. LITT-P) are of influence regarding interpretation of the data. Moreover, all the surgeries were performed by the same surgeon, but it is worth noting that the surgeon's personal learning curve during the earlier stages of his career where more SSI was conducted may play a role in the analyzed outcomes. At the beginning of the study period (March 2015), the surgeon was using the novel SSI technique as his personal standard approach. However, the standard approach in the overall field of BCD surgery at the time was the LITT-P technique. Eventually, the surgeon switched to the LITT-P approach from January 2017 onwards in order to conform with the standard approach in the BCD field. Also in this context, there may be a possible reporting bias of the Holgers score because of the variable follow-up periods after implantation.

Since follow-up analysis was limited, it would be beneficial to continue collecting data to identify long-term stability trends and soft tissue outcomes in combination with the inclusion of more patients. A future improvement is to conduct a prospective cohort study consisting of standardized follow-up lengths and randomization to eliminate potential bias and confounding factors, such as patient age, type of implant, and comorbidity.

## Conclusion

The present cohort study compares two implantation approaches to percutaneous BCDs. The outcomes reveal that the SSI could be an alternative option and promising innovation for patients based on its theoretical benefits in combination with the favorable soft tissue tolerability that was demonstrated in this study. However, increased cases of implant loss and operative duration in the SSI cohort warrant further investigation, since these relevant issues show more beneficial results in the LITT-P.

## Data Availability Statement

The raw data supporting the conclusions of this article will be made available by the authors, without undue reservation.

## Ethics Statement

The studies involving human participants were reviewed and approved by the Dutch Central Committee on Research Involving Human Subjects (CCMO) according to the Netherlands Medical Research Involving Human Subjects Act. Written informed consent for participation was not required for this study in accordance with the national legislation and the institutional requirements.

## Author Contributions

RS had full access to all the data, takes full responsibility for the integrity of the data, takes responsibility for the accuracy of the data analysis, conceptualized the study, performed the literature review, collected the data, drafted the article, and performed critical revisions of the article. SS compiled and analyzed the data, drafted the article, and performed critical revisions of the article. AB conceptualized the study, analyzed the data, and performed critical revisions to the article. HT and SD conceptualized the study, analyzed the data, and performed critical revisions of the article. All authors approved this study for publication. All authors contributed to the article and approved the submitted version.

## Conflict of Interest

The authors declare that the research was conducted in the absence of any commercial or financial relationships that could be construed as a potential conflict of interest.

## Publisher's Note

All claims expressed in this article are solely those of the authors and do not necessarily represent those of their affiliated organizations, or those of the publisher, the editors and the reviewers. Any product that may be evaluated in this article, or claim that may be made by its manufacturer, is not guaranteed or endorsed by the publisher.
